# Research on Lightweight Dynamic Security Protocol for Intelligent In-Vehicle CAN Bus

**DOI:** 10.3390/s25113380

**Published:** 2025-05-27

**Authors:** Yuanhao Wang, Yinan Xu, Zhiquan Liu, Suya Liu, Yujing Wu

**Affiliations:** 1 The College of Engineering, Yanbian University, Yanji 133002, China; davidwang0214@outlook.com (Y.W.); ynxu@ybu.edu.cn (Y.X.); 1224022474@ybu.edu.cn (S.L.); 2 The College of Cyber Security, Jinan University, Guangzhou 510632, China; zqliucom@gmail.com

**Keywords:** intelligent and connected vehicles, CAN bus, dynamic compression algorithm, dynamic key management, defense capability, dynamic encryption and authentication

## Abstract

With the integration of an increasing number of outward-facing components in intelligent and connected vehicles, the open controller area network (CAN) bus environment faces increasingly severe security threats. However, existing security measures remain inadequate, and CAN bus messages lack effective security mechanisms and are vulnerable to malicious attacks. Although encryption algorithms can enhance system security, their high bandwidth consumption negatively impacts the real-time performance of intelligent and connected vehicles. Moreover, the message authentication mechanism of the CAN bus requires lengthy authentication codes, further exacerbating the bandwidth burden. To address these issues, we propose an improved dynamic compression algorithm that achieves higher compression rates and efficiency by optimizing header information processing during data reorganization. Additionally, we have proposed a novel dynamic key management approach, incorporating a dynamic key distribution mechanism, which effectively resolves the challenges associated with key management. Each Electronic Control Unit (ECU) node independently performs compression, encryption, and authentication while periodically updating its keys to enhance system security and strengthen defense capabilities. Experimental results show that the proposed dynamic compression algorithm improves the average compression rate by 2.24% and enhances compression time efficiency by 10% compared to existing solutions. The proposed security protocol effectively defends against four different types of attacks. In hardware tests, using an ECU operating at a frequency of 30 MHz, the computation time for the security algorithm on a single message was 0.85 ms, while at 400 MHz, the computation time was reduced to 0.064 ms. Additionally, for different vehicle models, the average CAN bus load rate was reduced by 8.28%. The proposed security mechanism ensures the security, real-time performance, and freshness of CAN bus messages while reducing bus load, providing a more efficient and reliable solution for the cybersecurity of intelligent and connected vehicles.

## 1. Introduction

With the widespread adoption of 5th Generation Mobile Communication Technology (5G) networks, intelligent and connected vehicles represent the forefront of automotive technology, integrating intelligent mobile spaces with multifunctional application terminals. These vehicles not only connect to the external world via the internet but also utilize onboard sensors, controllers, and actuators to enable vehicle-to-everything (V2X) communication. This capability allows for collaborative decision-making and intelligent control, driving the advancement of autonomous driving technology [1,2].

**Figure 1:**
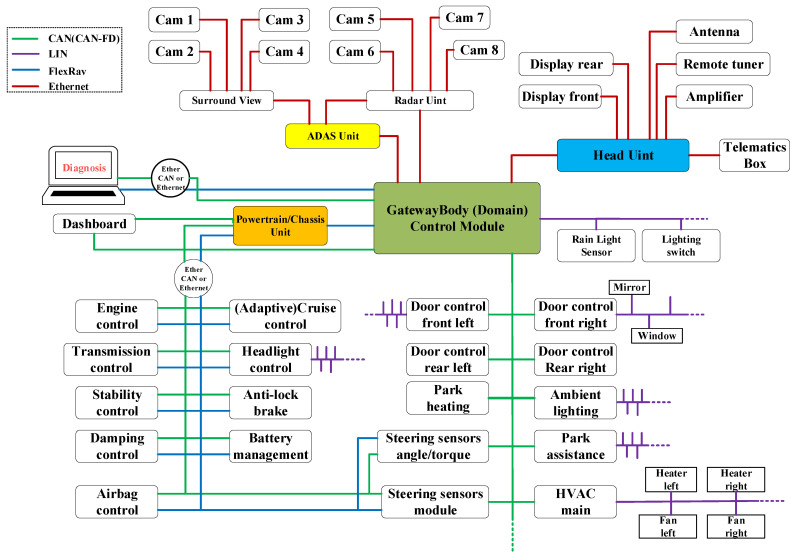
Diagram of bus connectivity in intelligent connected vehicles.

Intelligent and connected vehicles employ various bus protocols to build their internal communication systems, including CAN (and CAN with Flexible Data-Rate (CAN-FD)) [3], Local Interconnect Network (LIN), FlexRay, and Ethernet, as illustrated in Figure 1 [4]. Among these, the LIN is primarily used for low-speed data transmission in body and in-cabin components, such as air conditioning, doors, and lighting systems. The CAN bus (including CAN-FD), a high-speed and reliable bus system, is extensively applied in powertrain and safety systems, such as Electronic Control Units (ECUs) and anti-lock braking systems, as well as in instrument control and anti-theft systems. FlexRay, known for its high speed and reliability, is primarily utilized in advanced powertrain and chassis control systems [5]. Ethernet, on the other hand, meets the high-bandwidth data transmission demands between Advanced Driver-Assistance Systems (ADASs) and central control units, while also supporting high-speed communication for in-vehicle multimedia services.

Despite the diversity of these communication protocols, the CAN bus remains the most widely used. However, its built-in safety features primarily focus on communication reliability rather than network security [6].

In recent years, the increasing number of in-vehicle devices and external interfaces has highlighted the security vulnerabilities of the CAN bus. Messages on the CAN bus are typically transmitted in plaintext and lack authentication mechanisms to ensure message integrity. This allows attackers to infiltrate the in-vehicle bus network via external interfaces, enabling them to steal or even manipulate communication content. One of the most notable vehicle attacks occurred in 2015, where attackers successfully remotely controlled a moving car, manipulating its acceleration, braking system, and devices, such as the air conditioning, wipers, and radio. This incident led to the recall of 1.4 million vehicles by the manufacturer [7]. It exposed critical security flaws in the CAN bus, where attackers injected malicious messages to gain access and control over the smart vehicle. Due to the absence of authentication mechanisms in the CAN bus, ECUs accept and execute malicious messages from attackers without verification, making vehicles vulnerable to spoofing and forgery attacks. Consequently, implementing encryption and authentication schemes to enhance CAN bus security has become a crucial approach to improving its defense capabilities.

While existing security measures such as encryption, authentication, network isolation, and intrusion detection systems (IDSs) can enhance CAN bus security to some extent, each method has its limitations. Encryption and authentication schemes protect data confidentiality and access control but often increase system complexity and performance overhead, and they introduce challenges in key management. Network isolation can reduce the attack surface but may hinder system collaboration. IDSs can monitor bus activity but are prone to false positives and may incur high maintenance costs. Physical security measures like isolation, shielding, and protection can prevent physical access but may increase costs and weight, making them ineffective against sophisticated attacks.

We have analyzed and summarized the issues, providing corresponding solutions:

- Reducing bus load rate: The current load rate can still be reduced. We employ an improved dynamic compression module that optimizes the data compression algorithm to reduce the amount of transmitted data, thereby lowering the bus load rate.
- Static key management problem: Existing methods often rely on static keys, which cannot effectively meet the freshness requirements of keys in dynamic environments and are vulnerable to attacks. We have designed a new dynamic key-update mechanism that regularly updates keys to ensure their freshness and security while avoiding the risks associated with key expiration or leakage.
- System complexity and performance overhead: Traditional encryption and authentication mechanisms, while providing a certain level of security, often increase system complexity and degrade performance, making it difficult to meet the real-time requirements of in-vehicle networks. We have designed an improved real-time encryption and authentication mechanism that optimizes the encryption algorithm and authentication process, reducing the computational complexity and performance overhead of the system, ensuring efficient operation even under high load conditions.
- Lack of dynamic security strategies: Current research often focuses on single security measures, lacking a comprehensive consideration of the system’s lifecycle security, making it difficult to address complex attack scenarios. We propose to regularly update and upgrade security strategies, continuously monitoring and improving security measures from the design, manufacturing, to usage stages, ensuring that the system can respond to new security threats at different phases.

Therefore, to ensure the security of the CAN bus, it is essential to integrate multiple dynamic security measures, achieving a balance between system performance and security.

In Section 2, we provide a comprehensive overview of the technical aspects of in-vehicle CAN bus systems, delve into the current research landscape of CAN bus attacks, and systematically categorize the main types of CAN bus security protocols. These discussions lay a solid theoretical foundation for subsequent research. In Section 3, we address the inherent conflict between CAN data-frame security and compression efficiency by proposing a novel lightweight dynamic security protocol tailored for in-vehicle CAN bus systems. The proposed protocol not only ensures data security but also significantly enhances data compression rates. To thoroughly evaluate the performance of this system, we first collected CAN data from three different types of vehicles after a period of continuous operation, constructing a representative dataset. Subsequently, based on this dataset, we simulated various existing attack methods to conduct the comprehensive testing and validation of both the hardware implementation and software system of the proposed security protocol.

## 2. An Introduction to Information Security Technology for Vehicle Bus Networks

### 2.1. Introduction to In-Vehicle CAN Bus

In modern automotive electronic systems, the CAN bus plays a crucial role by connecting various ECUs and sensors through a shared communication network. This networked structure allows efficient data exchange between in-vehicle control units and sensors, enabling coordinated operations. Typically, low-speed CAN buses are used for simpler control tasks such as window adjustment and seat control, while high-speed CAN buses handle critical control information, such as engine management and braking systems. As the complexity of vehicle electronic systems continues to increase, issues related to communication security, network capacity, and communication conflicts are becoming more prominent, and these must be addressed to ensure the reliability and safety of the vehicle [8].

**Figure 2:**
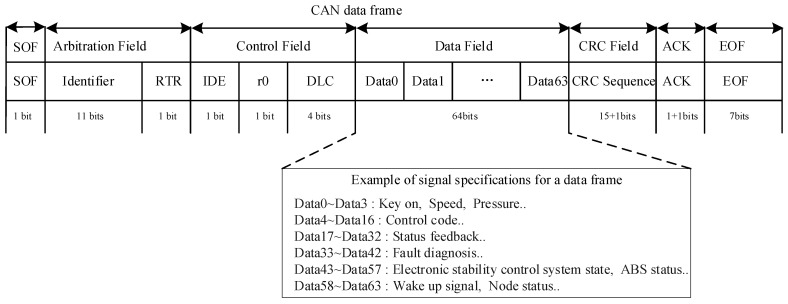
CAN 2.0A data-frame format diagram.

In the vehicle electronic system, the data frames transmitted over the CAN bus carry key control information, including sensor statuses, control commands, and feedback signals. ECUs transmit various types of information through the data field of the data frame (as shown in Figure 2), which typically includes the following categories of key data [9]:

- Sensor data: ECUs receive data from various sensors via the CAN bus, such as vehicle speed, engine Revolutions Per Minute (RPM), throttle position, temperature, pressure, and battery voltage. These data are transmitted in real time via the data field for the ECU to perform corresponding control and adjustments.
- Control commands: ECUs package control commands in the data field and send them to other modules. For example, the engine ECU may send commands via the CAN bus to the fuel injector control module to adjust the fuel injection amount or to the transmission control module to change gears.
- Status feedback: ECUs also send status information about themselves or feedback from other subsystems. For instance, the body control module may notify the lighting system’s operational status via the CAN bus, or the seat adjustment module may report the current seat position status.
- Fault diagnosis data: When issues occur in the vehicle, the ECU can send fault codes and diagnostic information through the CAN bus. This information is crucial for vehicle maintenance, as repair technicians can read the diagnostic information from the CAN bus to identify the source and nature of the fault.
- Safety-related information: Data frames also contain critical safety-related information, such as the operational status of the anti-lock braking system (ABS), the trigger state of the electronic stability control system, and signals for airbag deployment. This information is vital for ensuring timely responses in emergency situations.
- Network management information: ECUs also exchange network management information through the CAN bus, such as wake-up signals, sleep signals, and node statuses. This information helps manage the vehicle’s network power states, saving energy and ensuring the proper functioning of the system.

Automakers can customize the data fields to transmit different types of signals based on their specific needs. As illustrated in Figure 2, the CAN data frame begins with the Start-of-Frame (SOF), followed by the arbitration field, which consists of an 11-bit identifier (ID) and the Remote Transmission Request (RTR) bit. Subsequent to the arbitration field, the control field includes an Identifier Extension Bit (IDE), a reserved bit (r0), and a 4-bit Data Length Code (DLC), which encodes the number of data bytes. Following this is the data field, which carries the actual data and can range in size from 0 to 8 bytes. The remainder of the data frame comprises the Cyclic Redundancy Check (CRC) field, the Acknowledge (ACK) field, and the End-of-Frame (EOF). Our primary security measures are implemented within the data segment:

- Data 0–Data 3 (4 Bytes): Key on, speed signal, pressure, etc.;
- Data 4–Data 16 (13 Bytes): control information;
- Data 17–Data 32 (16 Bytes): status feedback information;
- Data 33–Data 42 (10 Bytes): fault diagnosis information;
- Data 43–Data 57 (15 Bytes): ABS status information;
- Data 58–Data 63 (6 Bytes): wake-up signals or node status information.

ECUs can efficiently exchange data through the CAN, which is a critical feature of modern automotive systems. However, in recent years, with advancements in automotive technology, the number of ECUs in vehicles has reached 80 to 100, and this number is expected to continue growing as vehicles become more electronic. As the number of ECUs and the volume of data exchange increase, the load on the network bus also rises, potentially causing delays in the transmission of critical information. Therefore, it has become particularly urgent to develop efficient data compression algorithms to address this challenge. Such algorithms will help optimize network performance, ensuring that critical information can be transmitted promptly and reliably, thereby supporting the development of more complex and intelligent automotive systems.

### 2.2. Research on CAN Bus Attack

With the increasing sophistication of vehicle network attack methods, the number of ECUs and gateways connected to in-vehicle bus systems has been growing significantly, thereby expanding the potential attack surface. Next, we will introduce some of the latest research advancements in CAN bus attacks:

Ying et al. [10], camouflage attack: A new camouflage attack method was proposed, where the attacker manipulates the transmission time of deceptive messages to simulate clock skew, thereby evading detection. The attack works by introducing delays, making it harder to detect the malicious behavior.

Hounsinou et al. [11], shutdown attack: A scheduling-based attack framework for CAN bus shutdown attacks was introduced. This framework takes advantage of the real-time scheduling characteristics of the CAN bus, predicting and exploiting additional attack opportunities, thereby increasing the vulnerability of the CAN bus.

Hau et al. [12], object removal attacks (ORAs): Based on Light Detection and Ranging (LiDAR) deception attacks, a more dangerous attack type was discovered—object removal attacks (ORAs). The goal of ORAs is to disable three-dimensional (3D) object detectors, which seriously threatens the performance of 3D target detection models in autonomous vehicles.

Iehira et al. [13], bus-off attack: A new deception attack method was proposed, capable of completely preventing the transmission of regular messages, with the ECUs failing to detect any errors. The attacker’s prototype was implemented using a Field-Programmable Gate Array (FPGA), showing that the deception message transmission rate could reach 100%.

Agbaje et al. [14], weeping CAN attack: A new zero-phase synchronization attack method was introduced. They identified a propagation strategy that enables the attacker to target more ECUs, extending the attack beyond the original expectations.

Serag et al. [15], vulnerability attacks: They developed the CAN Operation eXplorer (CANOX), an automated testing tool, to explore the impact of operations outside of the default error activity state. They discovered three major undiscovered vulnerabilities in the CAN standard, including the Single-Frame Bus-Off (SFBO) vulnerability, the Transmitter Error Counter (TEC) vulnerability in ECUs, and the persistent bus-off vulnerability. Combining these vulnerabilities, they created a Scan-and-Strike Attack (STS), a multi-stage attack that can quickly identify and disable critical ECUs in the vehicle’s CAN bus structure, even if the attacker knows nothing about the internal bus structure, and continuously prevent recovery.

The correlation analysis is shown in Table 1.

### 2.3. CAN Bus Security Protocol Type

To effectively defend against CAN bus communication attacks, researchers have developed various security protocols, which can be broadly classified into two categories: encryption- and authentication-based security protocols and gateway-based key-update security protocols.

#### 2.3.1. Encryption- and Authentication-Based Security Protocols

Woo et al. [16] scheme: A scheme that uses identity authentication codes to verify the source of CAN bus messages was proposed. This scheme uses Advanced Encryption Standard128 (AES-128) [17] encryption for each message and generates a 32-bit Message Authentication Code (MAC), which is divided into two parts and embedded into the CAN message’s extended ID and Cyclic Redundancy Check (CRC) fields. However, this method occupies the CRC field, preventing the original CRC from being performed. The key-update process is distributed through the gateway, which increases the bus load, although each update can be completed within 6 ms, resulting in a high time cost.

Palaniswamy et al. [18] performed a security analysis of Woo’s protocol and proposed a new protocol that includes entity authentication, key management, etc. They proved its security using the random oracle model and verified it with the Tamarin tool. Simulation results showed that while the protocol improved efficiency, frequent data exchanges still increased the bus load.

Michaels et al.’s [19] low-cost authentication mechanism: A low-cost message authentication mechanism was proposed that does not require large-scale modifications to existing CAN hardware and protocols. It adds additional Radio Frequency (RF) modules at both the sender and receiver ends to achieve message verification, but it adds extra cost.

Yu et al.’s [20] edge-computing-based security protocol: A new security protocol was proposed to address new vulnerabilities in edge computing and attribute-based access control. It uses hash functions, symmetric encryption, and other technologies to achieve fine-grained attribute-based encryption, but this increases the system’s overall latency and complexity.

Rasheed et al.’s [21] encryption-based subsystem: A subsystem was proposed that supports CAN authentication, data encryption/decryption, data traceability, and replay attack detection. Data confidentiality is achieved through lightweight block cipher authentication encryption, data traceability is enabled through blockchain methods, and anti-replay mechanisms were tested under different data infection rates.

Groza et al.’s [22] LiBrA-CAN: A lightweight broadcast authentication scheme called LiBrA-CAN was proposed. This scheme uses cryptographic techniques to generate and verify MACs to ensure message integrity and authenticity. A dual-layer authentication mechanism was designed by utilizing pre-shared keys and short-term keys to enhance security.

#### 2.3.2. Gateway-Based Key-Update Security Protocol

Jo et al.’s [23] centralized authentication method: A node-based centralized authentication method was designed to prevent camouflage attacks. All messages must undergo verification through an entity verification shared key named Auth, but this method carries the risk of a single point of failure.

Jo et al.’s [24] key-type selection: In in-vehicle network (IVN) security schemes, key types can be categorized into group keys, global keys, and pairwise keys. In the group-key-based scheme, ECUs are classified into different groups, with all ECUs in the same group sharing a unique key. However, if one ECU is compromised, all ECUs in the same group are at risk.

The correlation analysis is shown in Table 2.

### 2.4. Research on CAN Bus Data Compression Algorithm

Kim et al.’s [25] 4-bit data arrangement algorithm (4bit-DA) divides the 64-bit data field into 16 4-bit signals, calculates the rate of change in these signals, and rearranges them in order from large to small to generate 16 parts. Subsequently, these parts are filled into a 3 × 6 two-dimensional matrix from right to left and from top to bottom to form three reorganized signals. This algorithm effectively solves the data segmentation problem but fails to reduce the number of bytes actually sent, so there are certain limitations in compression efficiency.

Wu et al.’s [26] dynamic reconstruction compression (DRC) algorithm optimizes the next round of reorganization strategy according to the message content of the current round by dynamically adjusting the order of the reorganized signal. Compared with the 4bit-DA algorithm, DRC can omit the transmission of bytes with a change rate of zero, thereby significantly improving the compression efficiency. In addition, the algorithm achieves a stable compression rate by periodically transforming the reorganized signal. However, since the header bit used to identify the small signal data as zero is introduced in the signal reordering process, four additional bits need to be sent in each round of transmission, which increases the data transmission overhead to a certain extent.

The correlation analysis is shown in Table 3. Most existing schemes proposed in the literature have a significant impact on the load rate of the CAN bus. Although the scheme proposed in [19] has a relatively small impact on the bus load rate, it requires additional hardware costs. On the other hand, the scheme in [22], while having a modest impact on the bus load rate, faces challenges in key management due to its dual-layer authentication mechanism using pre-shared keys and short-term keys.

Based on the above analysis, existing in-vehicle CAN bus security protocols struggle to effectively balance dynamic key management, encryption/authentication mechanisms, and bus load efficiency. Several critical limitations can be identified: First, most current protocols support either encryption or authentication individually, making it difficult to achieve an efficient combination of both. Some schemes attempt to embed authentication codes into the CRC field or the extended identifier field. While this may save data space, it disrupts the original CAN frame structure and compromises compatibility and usability. Second, many security mechanisms require that each message undergo a complete process of encryption, authentication, and key update. This introduces significant computational overhead and negatively impacts real-time communication, particularly in high-frequency message transmission scenarios. Third, existing security designs lack effective strategies to reduce bus load. The transmission of additional encryption and authentication data often increases message length, leading to higher bus occupancy and, consequently, degraded overall communication performance in the vehicle network. Therefore, there is an urgent need to design a novel CAN bus security protocol with the following core features:

- Optimized encryption and authentication mechanisms to significantly reduce bus load;
- An efficient dynamic key management scheme that ensures secure communication while maintaining real-time performance with minimal resource overhead;
- Maximum compatibility with existing hardware architectures to avoid high system-upgrade costs.

Such an innovative protocol is expected to enhance the security and performance of in-vehicle networks while maintaining the economic feasibility and engineering practicality of system deployment.

## 3. Design of CAN Bus Dynamic Encryption Authentication System

Most existing security protocols are static and rely on Gateway Electronic Control Units (GECUs) for key management. This approach is not only costly but also lacks flexibility in key distribution. To address this issue, we propose a novel CAN bus security protocol that incorporates an improved dynamic compression module. This module increases redundancy in the data segment to embed authentication codes, ensuring that both the message and authentication code are transmitted within the same frame, thereby effectively reducing bus load.

To further mitigate the security risks associated with static keys, we designed an innovative key-update mechanism. At the beginning of each communication round, this mechanism generates a key seed using a custom nonlinear function, then dynamically produces keys using a Linear Feedback Shift Register (LFSR). These keys are combined with the count matrix of the DRC algorithm to update the plaintext in the encryption module. Since encryption and authentication share the same set of keys, this scheme eliminates the need for a dedicated key management mechanism. This not only reduces memory usage but also enhances the security performance of the CAN bus while minimizing communication latency.

**Figure 3:**
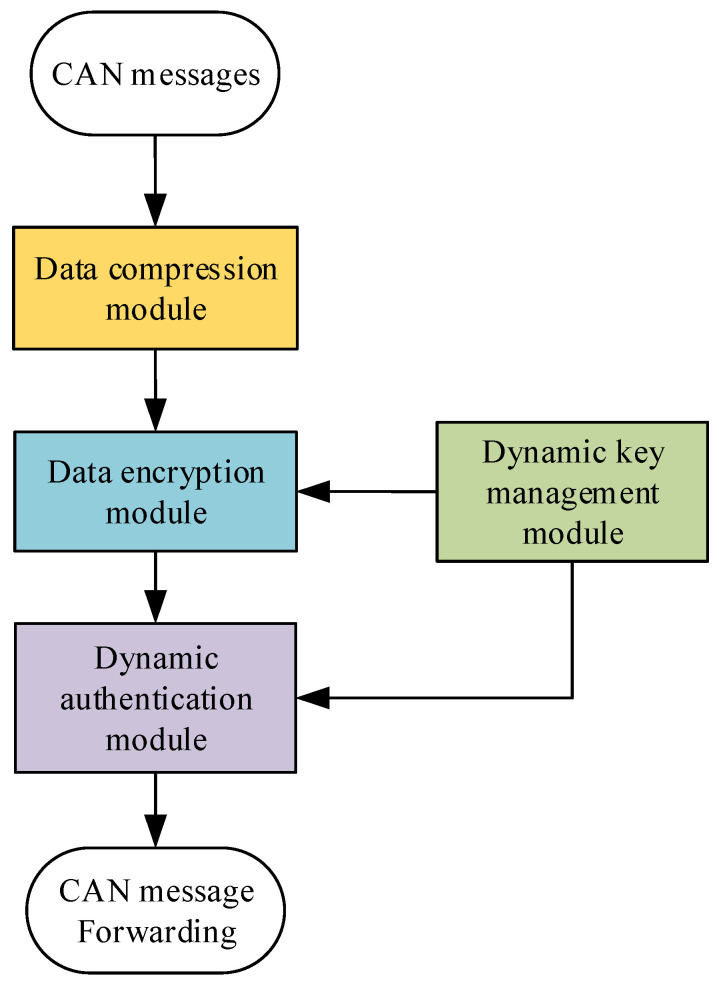
The security protocol structure diagram of the in-vehicle CAN bus.

The proposed CAN bus security protocol consists of four core modules: the data compression module, data encryption module, dynamic identity authentication module, and dynamic key distribution and management module, as illustrated in Figure 3. This solution ensures security while optimizing resource utilization and real-time performance.

The compression module is responsible for reducing the size of in-vehicle CAN bus data frames to fewer bytes, with the redundant portion being utilized to append authentication codes. For the dynamic key management module, we employ DBK (Dynamic Byte Key Generation and Management) to facilitate dynamic key generation, distribution, and administration. The data encryption component utilizes the AES-128 algorithm to encrypt the compressed data. Dynamic identity authentication is achieved through the hash-based message authentication code (HMAC) algorithm, which embeds authentication codes of varying lengths within the redundant space of the compressed data, depending on the degree of compression. This ensures the integrity and security verification of the data.

The compression module compresses in-vehicle CAN bus data frames. The data encryption module uses the AES-128 algorithm to encrypt data. The dynamic identity authentication module applies the HMAC algorithm to embed authentication codes of varying lengths into the redundant space of messages, depending on the compression level, to ensure data integrity and security.

### 3.1. Data Compression Module

As the number of ECUs in the car increases, the bus load rate rises, and the bandwidth resources are insufficient, it may lead to communication delays, data loss, and system performance degradation. To address the challenge of embedding authentication codes into redundant space while ensuring real-time message verification, we propose the Improved Dynamic Rearrangement Compression (IDRC) algorithm. This algorithm eliminates the 4-bit header during transmission to improve compression rates further and uses the sequential matrix of the compression module as plaintext in the key management module. This reduces memory usage, ensures the freshness of plaintext, and enhances system real-time performance and efficiency.

The IDRC algorithm consists of two main modules: the sequence confirmation module: generates the arrangement order for the next round of signals based on the messages in the current round. Signal rearrangement compression module: fills the rearrangement signal matrix with current data to ensure the efficiency and accuracy of the data compression process.

The mechanism operates in rounds of 100 messages. In each round, the first message transmits the original data to verify data integrity, ensure no information loss during compression, and provide a recovery point. This design enhances system robustness and facilitates debugging and error correction.

#### 3.1.1. Sequence Confirmation Module

1. Message decomposition: Equation (1) represents the process of decomposing each 64-bit message into 16 signals of 4 bits each.

(1) $${Message}_{n}=D_{n,1}D_{n,2}D_{n,3}\ldots D_{n,16}\;\left(1\leq n\leq 100,\;1\leq j\leq 16\right).$$

Here, $D_{n,j}$ represents the *j*-th 4-bit signal in the *n*-th message.

2. XOR calculation: For two consecutive messages, the process of calculating the XOR value of each signal is shown in Equation (2).

(2) $$D_{n,j}\left(xor\right)=D_{n-1,j}⨂D_{n,j}\;\left(2\leq n\leq 100\right).$$

where $D_{n-1,j}$ is the *j*-th signal of the previous message, and $D_{n,j}$ is the *j*-th signal ca of the current message.

3. Signal change counting: A counter tracks the change rate of each signal. If $D_{n,j}(xor)\neq 0$, the counter increases. The change rate is calculated as (3)
  (3) $$D_{j}\left(cnt\right)=\sum_{n=2}^{100}1_{\left(D_{n,j}\left(xor\right)\neq 0\right)}.$$
  where $1_{\left(D_{n,j}\left(xor\right)\neq 0\right)}$ is an indicator function that equals 1 if the condition $D_{n,j}\left(xor\right)\neq 0$ is true and 0 otherwise.

4. Sorting count results: The counters are sorted in ascending order as (4)

(4) $$sorted_{order}=sort\left(D_{1}\left(cnt\right),D_{2}\left(cnt\right),\ldots ,D_{16}\left(cnt\right)\right).$$

The next 100 messages are filled into the rearrangement signal matrix based on the sorted indices.

#### 3.1.2. Signal Rearrangement Compression Module

1. Matrix filling: initialization phase: The original sequence of 16 signals is filled into a 4 × 4 matrix sig as (5).

(5) $${sig}_{n}\left[m\right]\left[i\right]=D_{1+\left(m-1\right)*4}\;\left(1\leq m\leq 4,\;1\leq i\leq 4,\;1\leq n\leq 100\right).$$

An example of filling is as (6):

(6) $${sig}_{n}=\left[\begin{array}{cc} \begin{array}{cc} D_{1} & D_{5}\\ D_{2} & D_{6} \end{array} & \begin{array}{cc} D_{9} & D_{13}\\ D_{10} & D_{14} \end{array}\\ \begin{array}{cc} D_{3} & D_{7}\\ D_{4} & D_{8} \end{array} & \begin{array}{cc} D_{11} & D_{15}\\ D_{12} & D_{16} \end{array} \end{array}\right].$$

Subsequent rounds: The sorted indices from the previous round determine the sequence for filling sig. The filling rule, as shown in Equation (7), is to fill in from left to right, column by column.

(7) $${sig}_{n}\left[m\right]\left[i\right]=D_{sorted\text{\_}order[k]}\;\left(1\leq k\leq 16,1\leq m\leq 4,1\leq i\leq 4,1\leq n\leq 100\right).$$

where *sorted_order* is an array or list containing the indices of the signals sorted by the result of the signal change count. Its value is the original index of the signal, sorted from smallest to largest according to the counter value. *K* is the indicator of the sorted index and represents the position of the sorted signal in the original signal set. For example, if, after counter sorting, signal $D_{3}$ has the smallest rate of change, followed by $D_{1},D_{5},\ldots ,D_{8}$, then the *sorted_order* array could be [3, 1, 5,…, 8], so $D_{sorted\text{\_}order\left[1\right]}$ is $D_{3}$ and $D_{sorted\text{\_}order[16]}$ is $D_{8}$. This allows us to fill the signal into the recombination signal matrix according to the sorting result of the change rate. The recombination signal *sig*_12_ of the 12th message is shown in the following matrix (8).

(8) $${sig}_{12}=\left[\begin{array}{cc} \begin{array}{cc} D_{3} & D_{10}\\ D_{1} & D_{11} \end{array} & \begin{array}{cc} D_{16} & D_{4}\\ D_{9} & D_{6} \end{array}\\ \begin{array}{cc} D_{5} & D_{12}\\ D_{7} & D_{15} \end{array} & \begin{array}{cc} D_{2} & D_{14}\\ D_{13} & D_{8} \end{array} \end{array}\right].$$

2. XOR value calculation: For consecutive messages, the XOR values of all columns in each row of the sig matrix are calculated to generate a new 4-bit signal sequence, as shown in Equation (9).

(9) $${sig}_{k}\left(xor\right)\left[m\right]={sig}_{k-1}\left[m\right]⨂{sig}_{k}\left[m\right]\;\left(1\leq m\leq 4,\;2\leq k\leq 100\right).$$

3. Message matrix filling for transmission: The XOR values are directly filled into the message matrix without allocating header bits. The filling starts from bit 0, proceeding column by column until the last 1 appears, reducing execution time and improving data transmission efficiency.

**Figure 4:**
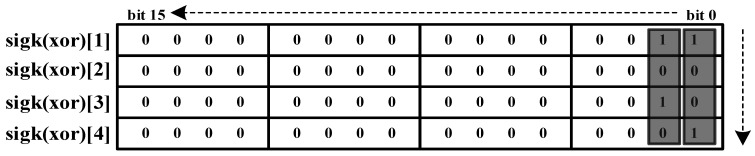
Recombination signal matrix.

For example, in Figure 4, given XOR values “11”, “00”, “10”, and “01”, the first column “1001” is filled into Figure 4 bits 0–3, and the second column “1010” is filled into bits 4–7. Using this method, the original 64-bit message compresses into just 8 bits for CAN bus transmission, as seen in Table 4.

### 3.2. Data Encryption and Authentication Module

#### 3.2.1. Data Encryption Module

Under the current conditions, cracking a 16-byte AES-128 encryption would take billions of years at the very least. Therefore, in the encryption module, we have adopted the AES-128 encryption algorithm. The plaintext data come from the sorting results generated by the compression module from the previous round, and the key is dynamically generated and provided by the key-update module. The compressed message output by the compression module is then XORed with the encrypted data generated by the encryption module, resulting in the final encrypted output. This design not only ensures the efficiency and security of data encryption but also significantly improves the system’s resistance to attacks by applying dynamic keys.

The plaintext is provided by the sorted order (*sorted_order*) generated by the compression module. The key is the one generated by the dynamic key-update module ($K_{l}$) (the initial keys for both the transmitter and receiver are preloaded by the program developer during system initialization to ensure key consistency between the two ends), and the result of the AES-128 algorithm is denoted as ${EM}_{l}$. The formula is as (10):

(10) $${EM}_{l}={AES}_{128}\left(K_{l},sorted\text{\_}order\right)\left(1\leq l\leq 50\right).$$

Since each round of processing involves 100 messages, we need to plan the message-handling process based on the worst-case scenario. In the worst case, the system needs to generate 50 AES-encrypted messages (considering that the AES-128 encryption algorithm produces 16-byte encrypted data, while our raw data may all be 8 bytes in size). Therefore, the system needs to prepare 50 independent AES encryption keys. Meanwhile, the *sorted_order* sequence generated by the compression module will be processed as the plaintext data for encryption. During the encryption process, the system will use the *l*-th key from the key group as the current encryption key.

The length of the compressed message varies, and during each encryption operation, the appropriate number of bytes from the generated ${EM}_{l}\;$ are XORed based on the byte size of the compressed message. This method significantly reduces the amount of encryption calculations and the frequency of key updates, improves the overall efficiency, and ensures the security of data.

The final encrypted result is calculated by performing an XOR between the compressed message ${CM}_{n}$ and the encryption result ${EM}_{l}$, as shown in (11):

(11) $${CT}_{n}=\left\{\begin{array}{c} {CM}_{n}⨁{EM}_{l}\left[\left(offset+1):(offset+len\left({CM}_{n}\right)\right)\right]\;\;\;\\ offset+len\left({CM}_{n}\right)\leq 16\;(1\leq l\leq 50),\\ {CM}_{n}\left[1:rem\right]⨁{EM}_{l}\left[(offset+1):16\right]||{CM}_{n}\left[(rem+1):len\left({CM}_{n}\right)\right]⊕{EM}_{l+1}\left[1:(len\left({CM}_{n}\right)-rem)\right]\;\\ offset+len\left({CM}_{n}\right)>16\;(1\leq l\leq 50) \end{array}\right..$$

In the encryption process, ${CT}_{n}$ represents the final encrypted result of the *n*-th message, ${len(CM}_{n})$ denotes the length of the *n*-th compressed message (in bytes), *offset* indicates the number of bytes already used in the current ${EM}_{l}$ (initialized to 0), rem = 16 − *offset* represents the number of remaining available bytes in the current ${\;EM}_{l}$, and || denotes the concatenation operation. When the remaining bytes in ${EM}_{l}$ are sufficient to process the entire ${CM}_{n}$, we use the first formula in Equation (11) for computation. When the remaining bytes in ${EM}_{l}$ are insufficient to process the entire ${CM}_{n}$, we split ${CM}_{n}$ into two parts: the first part is XORed with the remaining bytes of ${EM}_{l}$, and the second part is XORed with ${EM}_{l+1}$. Finally, the two parts are concatenated to form the final ${CT}_{n\;}$, as described in the second formula of Equation (11).

#### 3.2.2. Data Authentication Module

We use the HMAC-SHA-256 algorithm as the authentication method for the in-vehicle CAN bus network security protocol [27].

The plaintext data are provided by the sorting result *sorted_order* generated by the compression module. The keys are provided by the key group $K_{h}$ generated by the dynamic key-update module, where $K_{h}$ represents the *h*-th key in the key group used for computation. The HMAC-SHA-256 algorithm generates 32 bytes of authentication data in a single operation. In the worst-case scenario of processing 100 messages (assuming each message is 8 bytes in size), we need a maximum of 25 keys to generate 25 MAC authentication data. The result generated by the algorithm ${AM}_{h}$ is shown in Formula (12):

(12) $${AM}_{h}={SHA}_{256}\left(K_{h},sorted\text{\_}order\right)\;\left(1\leq h\leq 25\right).$$

Since the maximum data-frame length of the CAN bus is 8 bytes, the authentication data must also adhere to this limit. To improve the efficiency of the authentication process and meet real-time requirements, we have designed a dynamic identity authentication mechanism that adapts to the CAN bus data transmission rate.

Authentication efficiency analysis: The time *T* required to perform a brute-force attack on the standard CAN bus access is shown in Equation (13).

(13) $$T\left(s\right)=\frac{2^{B}}{R*F}.$$

where *B* is the number of bits of authentication code, *R* is the transmission rate of the CAN bus, and *F* is the maximum number of bits that can be transmitted in one frame. If the rate of the CAN bus is 1 Mbps, it will take 8.39 s to brute force the authentication code of 2 bytes after sending a message with a 128-bit frame including bit stuffing. If the authentication code selects 1 byte under the same conditions, the brute-force attack time is 0.0327 s. Therefore, we choose the authentication code of 2 or more bytes to be placed in the redundant position after encryption, which improves the security and does not need to place the authentication code on the CRC and extended ID to cause the normal work of CRC like [16,27].

We balance real-time performance and security by using two different methods to load the authentication code, depending on the size of the compressed and encrypted data:

When the size of the compressed and encrypted data is less than or equal to 5 bytes, we choose to place a 2-byte authentication code in the redundant position of the encrypted data. Our goal is to differentiate between compressed and uncompressed information, and we have selected 5 bytes as the threshold. By adding a 2-byte authentication code to the compressed message, it is possible to send both the encrypted and authenticated message within a single frame. This not only effectively enhances security but also avoids the serious issues that might arise from placing the authentication code in the CRC or extended ID.

The formula for the final message *mes* is as (14):

(14) $${mes}_{n}={CT}_{n}∥Extract\left({AM}_{h},2i-1,2\right)\;\left(1\leq i\leq 128\right)\;\left(1\leq h\leq 25\right).$$

Here, *Extract* is the extraction function. Since each operation only requires extracting 2 bytes of authentication code from the 256-bit (32-byte) data of ${AM}_{h}$ and appending it to ${CT}_{n}$ to form the final message, and the extraction index *i* ranges from 1 to 128. The extraction process starts from ${AM}_{1}$, where 2*i* − 1 is the starting position for extraction, and 2 indicates the length of extraction (i.e., 2 bytes). When all 256 bits of ${AM}_{1}$ are used, the system continues to extract bytes from ${AM}_{2}$ for authentication purposes, and so on.

When the compressed and encrypted message size is greater than 5 bytes, to ensure the CAN bus frame’s transmission safety, we send two frames. The first frame is the 8-byte encrypted message. The second frame contains the 8-byte authentication code.

The formula for the final message is as (15):

(15) $${mes}_{n,1}={CT}_{n},\;{mes}_{n,2}=Extract\left({AM}_{h},8i-7,8\right)\;\left(1\leq i\leq 32\right)\;\left(1\leq h\leq 25\right).$$

Here, *Extract* is the extraction function. Since in this case we need to extract 8 bytes of authentication code from the 256-byte data of ${AM}_{h}$, the extraction index i ranges from 1 to 32. The extraction process starts from ${AM}_{1}$, where 8*i* − 7 is the starting position for extraction, and 8 indicates the length of extraction (i.e., 8 bytes). When all 256 bytes of ${AM}_{1}$ are used, the system continues to extract bytes from ${AM}_{2}$ for authentication purposes, and so on.

#### 3.2.3. Dynamic Key Management Module

The AES algorithm is a symmetric encryption technology that requires both plaintext and a key for its operation. Similarly, HMAC operations also necessitate the involvement of a key. Therefore, the storage and protection of keys are central to ensuring the overall security of the system. In security protocols, the methods of key generation and renewal, as well as how the communicating parties synchronize symmetric keys, are critical points of focus.

In some current security protocols, key updates typically rely on a gateway. This method requires multiple communications between the ECU and the gateway. After receiving the key, the ECU must also calculate the remaining key chain. In practical applications, due to the large number of vehicle ECUs, this approach not only significantly increases the bus load but also wastes the computational resources of the ECUs, thereby reducing the overall efficiency and real-time performance of the system.

**Figure 5:**
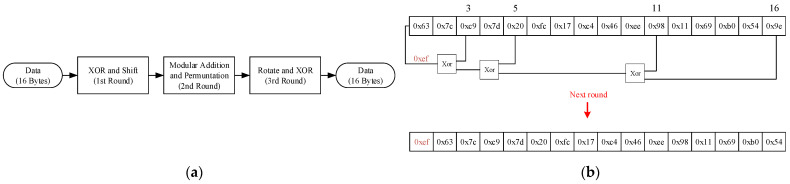
(**a**) Nonlinear function flow chart; (**b**) LFSR flow chart.

1. Key-Update Mechanism: To address the issues of high key management overhead, slow update speeds, and limited resources in traditional solutions, this paper designs a dynamic key generation, distribution, and management module named DBK (Dynamic Key Generation and Management). The DBK module automatically generates keys through the correct processing of the initially agreed-upon protocol by the sender and receiver, without the need for additional gateway communication support, thereby further reducing the bus load pressure and enhancing system efficiency and real-time performance. The DBK module is based on an LFSR for generating and managing the keys required for encryption and authentication, and its working principle is as follows:
  1.1. Initial Key Setup: During each round of key generation, our custom-designed nonlinear function first produces a 16-byte initial key value, as shown in Figure 5a.
  1.2. Dynamic Key Generation: Following predefined security policies, the system selects 4–6 specific bytes (including the least significant byte) to participate in subsequent XOR operations. The 16-byte key is shifted right by one unit as a whole, with the least significant unit being discarded. The pre-selected bytes are then subjected to an XOR operation one by one, and the result is filled into the most significant unit. Each shift generates a new 16-byte key, ultimately forming a key group for dynamic updates, as shown in Figure 5b.

For example, in Figure 5, after generating through nonlinear function, the initial key for both the sender and receiver is set to 0x63, 0x7C, 0xC9, 0x7D, 0x20, 0xFC, 0x17, 0x C4, 0x46, 0xEE, 0x98, 0x11, 0x69, 0xB0, 0x54, 0x9E. By selecting the 3rd, 5th, 11th, and 16th byte units for the XOR operation, the result 0xEF is obtained. The new key then becomes 0xEF, 0x63, 0x7C, 0xC9, 0x7D, 0 x20, 0xFC, 0x17, 0xC4, 0x46, 0xEE, 0x98, 0x11, 0x69, 0xB0, 0x54. Through this cyclic process, a key group is generated for encryption and authentication purposes. The DBK module enhances security by pre-configuring the XOR byte selection at the manufacturing stage, eliminating the need to transmit keys alongside data. This approach removes the requirement for a standalone Key Management Unit (KECU), thereby saving ECU memory resources. Additionally, since the keys do not occupy data-frame space, there is no additional communication overhead, significantly improving real-time performance and system adaptability.

### 3.3. Receiver Process Design

**Figure 6:**
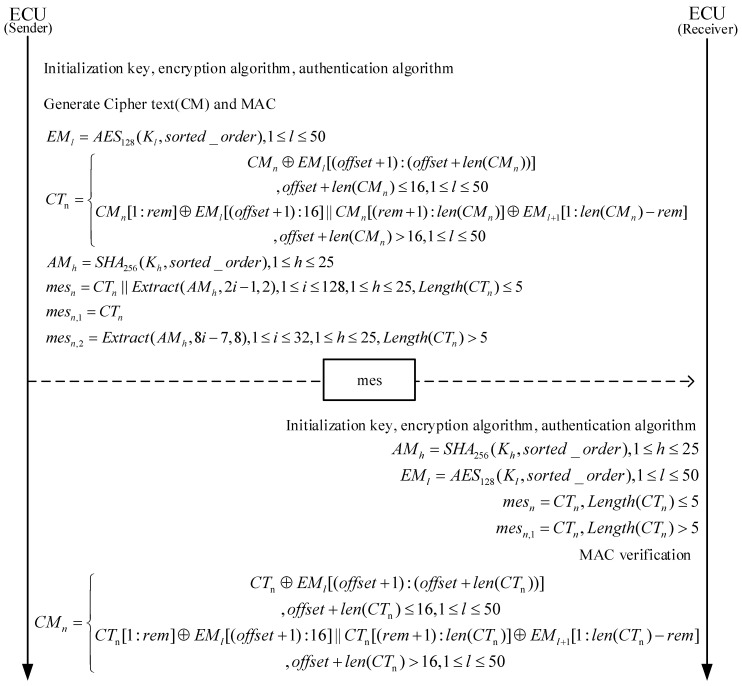
Sender and receiver processes.

The decoding steps on the receiver side are as Figure 6.

- Initialization: Upon startup, the receiver initializes the encryption and authentication algorithms and sets the initial key, which is the same as the initial key of the sender, ensuring that both parties can correctly decrypt and authenticate the message.
- Key Generation: The receiver uses the DBK module to dynamically generate the key based on the received message content. This requires the receiver to identify the data portion used for key generation.
- Message Length Determination: Based on the received message length, the receiver determines the length of the authentication code. If the message length is less than or equal to 7 bytes, the authentication code is 2 bytes; otherwise, the authentication code is 8 bytes.
- Authentication Code Verification: The receiver uses the HMAC-SHA-256 algorithm and the rearranged signal to generate the expected authentication code and compares it with the received authentication code to verify the integrity and authenticity of the message.
- Extract Authentication Code and Encrypted Data: The receiver extracts the authentication code and the encrypted data from the received message.
- Data Decryption: Using the AES-128 algorithm and the generated key, the receiver decrypts the encrypted data to obtain the compressed signal.
- Signal Rearrangement: The receiver rearranges the decrypted compressed signal according to the sender’s rearrangement logic to restore the original signal sequence.
- Data Decompression: If the authentication is successful, the receiver uses the corresponding decompression algorithm to decompress the signal and restore the original message. If authentication fails, the message is discarded and will not be transmitted onto the bus.

### 3.4. Bus Fault-Handling Solution

When a CAN bus communication failure (typically a physical layer fault) occurs, the CAN controller switches the faulty node from an error-active state to a bus-off state to prevent interference with normal nodes. Upon entering the bus-off state, the ECU is prohibited from affecting the bus in any way, including transmitting messages, ACKs (acknowledgment frames), error frames, or overload frames.

The CAN bus design specification strictly defines the self-recovery mechanism for bus-off nodes: the ECU must execute a recovery procedure after entering bus-off mode until the fault is resolved. This mechanism accounts for both transient and persistent faults, with a slow recovery phase lasting 160 ms.

If a bus-off state is triggered at any point in the security protocol, the built-in counter suspends incrementing. Once the bus resumes normal operation, the counter continues until it reaches 100 messages. Consequently, the message sequencing and data compression functions of the security protocol remain unaffected by bus-off states.

## 4. Experiment and Analysis

### 4.1. Experimental Data Source and Testing Environment

**Figure 7:**
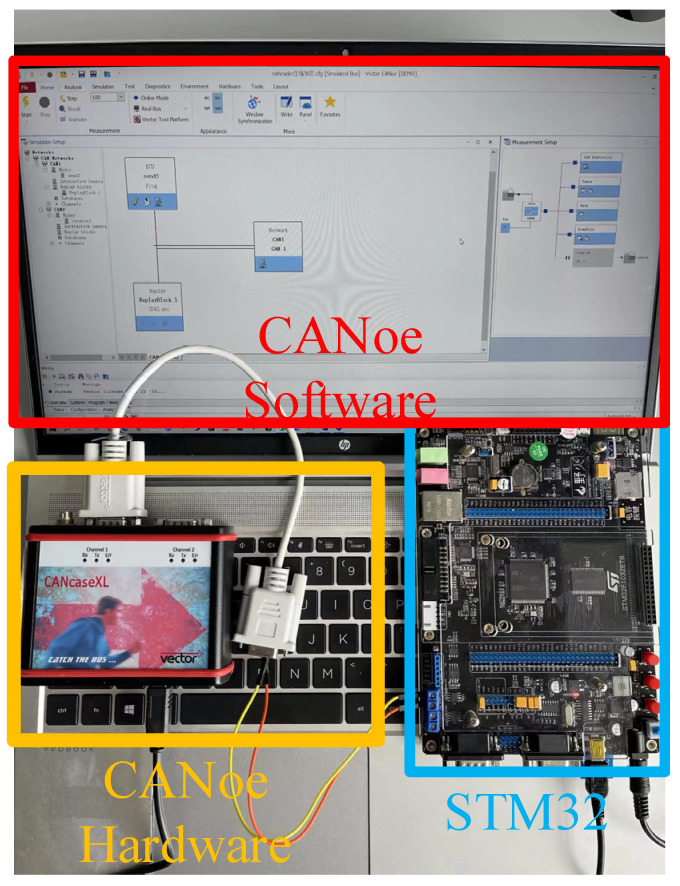
Experimental hardware diagram.

We extracted CAN data from Kia, Honda, and Chevrolet vehicles after they had been driven for some time to form the dataset. The proposed security method requires each ECU to independently calculate, generate, and allocate its own key. To simulate ECUs within different frequency ranges in the vehicle, we used STM32H743 and STM32F407 development boards from EmbedFire Company (Dongguan, China) to verify the practical feasibility of this security protocol. To evaluate the defense capabilities of the security protocol, we simulated several attack scenarios using CANoe 18.0.185 -64bit and verified the protocol’s effectiveness through actual attack and defense tests, as shown in Figure 7.

In the hardware testing environment, the usage of memory, flash memory, and Random-Access Memory (RAM) is summarized in Table 5. The program’s Read-Only MSemory (ROM) size is 36,697 bytes. During the program’s execution, the random read–write memory usage is 36,421 bytes. The program uses 36,884 bytes of flash memory. The proposed security protocol is able to run effectively within the limited in-vehicle storage resources.

### 4.2. Attack and Defense Results

We simulated four types of attacks in the CANoe software, eavesdropping attack, brute-force attack, replay attack, and flood attack, to evaluate the defense capabilities of the proposed security protocol.

#### 4.2.1. Eavesdropping Attack

Verification objective: to validate whether the proposed security protocol can effectively prevent attackers from obtaining meaningful CAN message content through eavesdropping.

An eavesdropping attack refers to an attacker accessing the in-vehicle CAN bus and stealing raw message data. Since the CAN bus uses a broadcast mechanism, any device connected to the interface could potentially listen to data transmission on the bus. Without encryption protection, attackers can reverse engineer the CAN messages to obtain the message IDs, data fields, and signal definitions, allowing them to steal CAN messages for the purpose of acquiring vehicle communication protocols. This can lead to replay attacks, data manipulation, or identity forgery, which disrupts the vehicle’s normal operation, leaks sensitive information, or enables remote control. Stealing messages provides the necessary information for subsequent malicious attacks, posing a severe threat to vehicle security.

**Figure 8:**
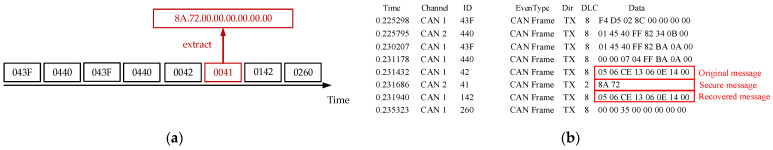
(**a**) Eavesdropping attack node transmission graph; (**b**) results of eavesdropping attack and defense.

In Figure 8, the “Original Message” displays unprotected CAN data, which attackers can directly read upon successful interception. In contrast, the “Secure Message” represents CAN messages processed by the security protocol. After compression, the message is reduced to 0 bytes, with an additional 2-byte authentication code appended. Only 2 bytes of data, “8A 72”, need to be transmitted, as shown in Figure 8a. When attackers extract data with ID 42, they obtain meaningless information. This experiment aims to verify whether the encryption and authentication mechanisms can effectively prevent eavesdropping attacks and ensure data integrity and security, as illustrated in Figure 8b. By comparing the “Original Message” and the “Secure Message”, we observe that messages processed by the security protocol effectively prevent attackers from obtaining meaningful data, thereby thwarting subsequent malicious attacks. The experimental results demonstrate that with the implementation of encryption and authentication mechanisms, attackers cannot easily access or tamper with the data, confirming the effectiveness of the proposed security protocol.

#### 4.2.2. Brute-Force Attack

Verification objective: to validate whether the encryption and authentication mechanisms can effectively resist brute-force attacks and ensure the security of CAN bus data.

The core idea of a brute-force attack is for the attacker to repeatedly send different message IDs or contents in an attempt to gather the desired information or disrupt normal communication. On the CAN bus, due to the lack of an authentication mechanism, any device connected to the bus can send messages. The attacker sends possible message IDs, starting from 0 × 000 and incrementing by one. The vehicle’s ECUs will perform related actions based on the received message IDs (e.g., unlocking doors and displaying vehicle speed). Although most of these attack messages will not be received by the corresponding ECUs due to mismatched IDs, a small number of anomalous messages with matching IDs might trigger behaviors similar to replay attacks, posing potential risks to the system.

**Figure 9:**
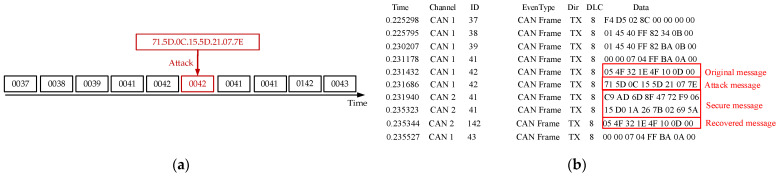
(**a**) Brute-force attack node transmission graph; (**b**) results of brute-force attack and defense.

In Figure 9, the “Original Message” displays unprotected CAN data, where attackers can send different message IDs through brute-force attacks until they intercept useful data. In Figure 9a, the “Attack Message” demonstrates how attackers can tamper with the data of ID 42, causing data corruption and occupying the bus channel. In the experiment, we applied the security protocol to the “Original Message”, transforming it into a “Secure Message” that includes encrypted data and an authentication code. The results in Figure 9b show that even when attackers send different message IDs through brute-force attacks, they cannot decrypt or verify the “Secure Message”, thus preventing them from accessing or tampering with the data. The restored message at the receiving end matches the original message, confirming the integrity and security of the data and validating the effectiveness of the security mechanism.

#### 4.2.3. Replay Attack

Verification objective: to determine whether the proposed security protocol can distinguish between attacker commands and normal commands.

**Figure 10:**
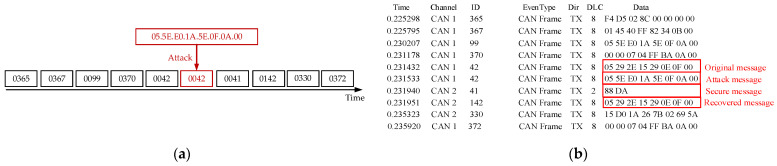
(**a**) Replay attack node transmission graph; (**b**) results of replay attack and defense.

Attackers can eavesdrop on legitimate communications on the bus, record messages with control functions, and replay these messages at a later time to forge control commands, deceiving ECUs into performing improper operations. As shown in Figure 10a, attackers attempt to bypass security mechanisms or cause system anomalies by replaying previously captured messages. In Figure 10b, when the receiver node receives an “Attack Message”, it performs HMAC authentication. If the MAC value of the replayed message does not match the MAC value calculated by the receiver, the replayed message will be discarded, preventing the execution of forged commands. For messages that pass authentication, the receiver’s “Secure Message” will be successfully decrypted through the security protocol and restored as the “Recovered Message”, ensuring data integrity and security. This process demonstrates that using authentication mechanisms like HMAC can effectively prevent replay attacks and safeguard the communication system.

#### 4.2.4. Flood Attack

Verification objective: to determine whether the proposed security protocol can ignore attacker commands and ensure the normal operation of the system.

**Figure 11:**
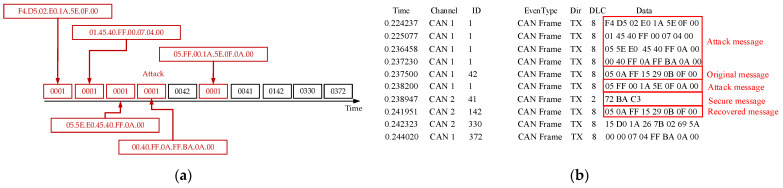
(**a**) Replay attack node transmission graph; (**b**) results of flood attack and defense.

The core idea of a flood attack is for the attacker to continuously send high-priority messages to the CAN bus, occupying bus resources, leading to a significant increase in bus load, which prevents or delays the transmission of other legitimate messages, especially low-priority ones, and interferes with the system’s normal operation, as shown in Figure 11a.

Figure 11b shows the specific process of a flood attack: The attacker continuously sends a large number of low-priority “Attack Message” to the CAN bus. These attack messages’ IDs and HMACs typically fail the security protocol’s validation mechanism. Since these messages fail the validation, they are discarded. For legitimate “Original Message”, their IDs and HMACs are validated through the security protocol. Once validation is successful, the messages are decrypted, restored to the “Recovered Message”, and can continue transmission and participate in subsequent network communication. The results demonstrate that the security protocol can effectively identify and discard attack messages while ensuring the transmission of legitimate messages, thereby validating its effectiveness.

In each experimental section, we clearly outlined the specific ideas to be verified and demonstrated the effectiveness of the proposed security protocol in addressing various attacks through experimental results. These findings indicate that the proposed security protocol can effectively prevent eavesdropping attacks, brute-force attacks, replay attacks, and flooding attacks, ensuring the security and reliability of in-vehicle network communications.

### 4.3. DBK Key Generation Time Comparison

In the proposed security protocol, each ECU independently generates, manages, and updates its own key, and the key-update time does not change as the number of ECUs increases. Since key generation and distribution are localized and depend on each ECU’s computing resources, there is no need for cooperation or synchronization with other ECUs. Even with more ECUs in the system, each ECU only processes its own key, keeping the key-update time constant. This method enhances system independence and flexibility.

**Figure 12:**
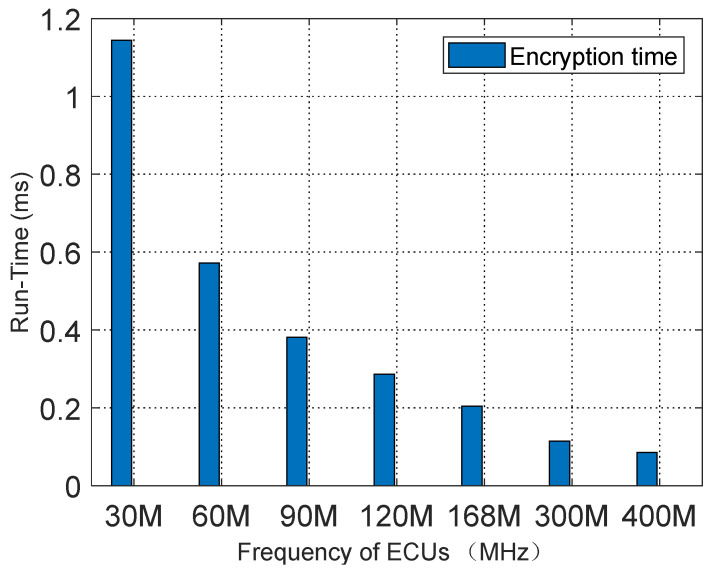
DBK update time.

We tested the DBK key generation time at various ECU frequencies. The experiment simulated multiple ECUs operating at frequencies of 30 MHz, 60 MHz, 90 MHz, 120 MHz, 168 MHz, 300 MHz, and 400 MHz. The experimental results, shown in Figure 12, demonstrate that when the ECU frequency is 30 MHz, the key generation time is 1.144 ms, whereas at 400 MHz, the execution time is reduced to 0.0858 ms.

To further verify the advantages of our scheme, we compared it with the schemes proposed by [23,27], as shown in Table 6. Compared to other schemes, our approach has significant advantages, especially in not relying on a KECU to store keys but instead using predefined rules to independently update keys. This greatly reduces memory and computation costs on the ECU. Specifically, compared to other schemes, our key generation time is only 0.0858 ms, demonstrating higher efficiency and lower latency. Table 6 shows the performance comparison, highlighting the significant advantages of our scheme in key generation time. These experimental results indicate that our scheme is highly competitive in terms of performance and cost, making it suitable for widespread use in automotive ECUs.

### 4.4. Security Protocol Runtime Analysis

**Figure 13:**
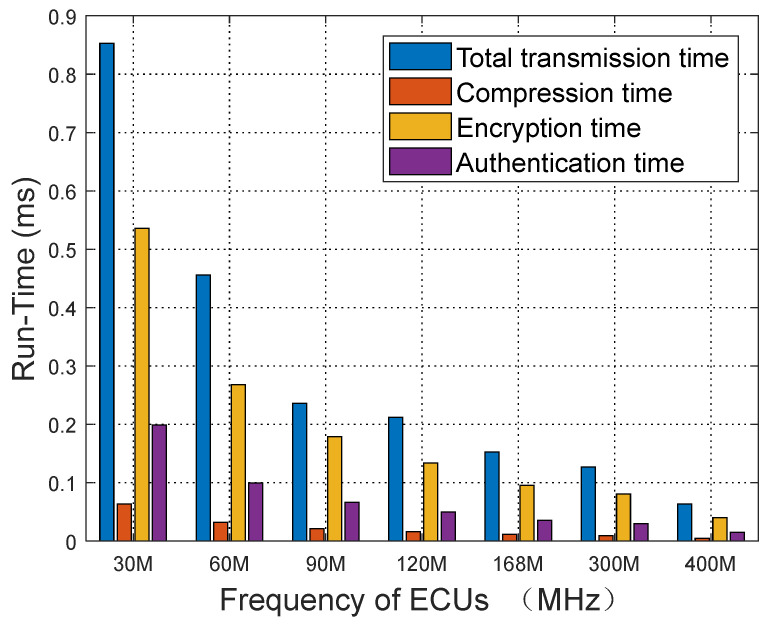
Execution time of each module.

Figure 13 illustrates the hardware execution times for each module of our security protocol at different frequencies, with data based on the processing time for 100 messages per round. At a frequency of 30 MHz, the execution times for the protocol modules are as follows: the total execution time is 0.85 ms, the AES-128 algorithm execution time is 0.54 ms, and the time to add the 2-byte MAC is 0.19 ms. In contrast, at a frequency of 400 MHz, the total protocol execution time is reduced to 0.064 ms, the AES-128 encryption time decreases to 0.04 ms, and the MAC addition time drops to 0.015 ms. These results demonstrate that even at lower ECU frequencies, such as 30 MHz, the protocol modules can operate efficiently without significant execution delays. This validates that our security approach can be implemented on low-frequency hardware in vehicles, meeting the requirements for real-time performance.

#### 4.4.1. Bus Load Rate Analysis

The load rate of the in-vehicle bus refers to the degree of bus data occupancy, usually represented as a percentage. It measures the ratio between data transmission frequency and total bandwidth. An excessively high load rate can lead to bus congestion, affecting the real-time nature of data transmission, potentially causing message delays or loss. The bus load rate is closely related to the compression result because the compression algorithm reduces the size of the data, directly decreasing the amount of data transmitted over the bus, thereby alleviating the bus burden.

#### 4.4.2. Compression Effect and Execution Time Comparison

**Figure 14:**
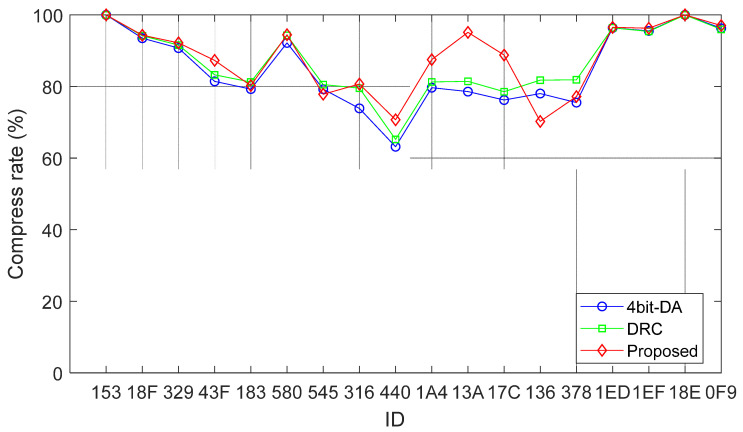
Compression effect comparison chart.

We compared the compression ratios of the proposed in-vehicle bus data compression algorithm and the DRC algorithm on CAN bus messages with different IDs using the experimental dataset. As shown in Table 7, a total of 1,237,315 CAN bus messages with different IDs were tested. Compared with the 4bit-DA, the proposed algorithm achieved a 7.52% higher compression ratio on ID 440. On average, the proposed algorithm achieved a 2.90% higher compression ratio when compared with other ID data. Compared with the DRC algorithm, the proposed algorithm achieved a 5.46% higher compression ratio on ID 440. On average, the proposed algorithm achieved a 1.10% higher compression ratio when compared with other ID data. These results demonstrate the proposed algorithm’s advantage in improving compression efficiency, as shown in the line chart in Figure 14.

**Figure 15:**
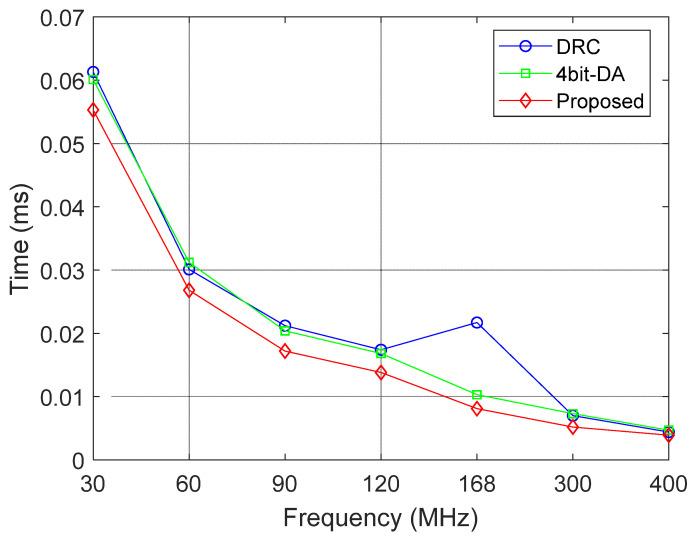
Compression time comparison chart.

Table 8 compares the hardware execution time of the DRC algorithm and the proposed algorithm at different frequency bands, including both compression and decompression processes. At the 30 MHz frequency band, the compression time is 0.0553 ms, and the decompression time is 0.0892 ms. At the 400 MHz frequency band, the compression time is significantly reduced to 0.0039 ms, and the decompression time is 0.00656 ms. Compared with the 4bit-DA algorithm, the proposed algorithm reduces the compression time at 30 MHz (0.0613 ms) by approximately 8%. Compared with the DRC method, the proposed algorithm reduces the compression time at 30 MHz (0.0613 ms) by about 10%. These results demonstrate higher efficiency, as shown in the line chart in Figure 15.

#### 4.4.3. Load Rate Comparison

**Figure 16:**
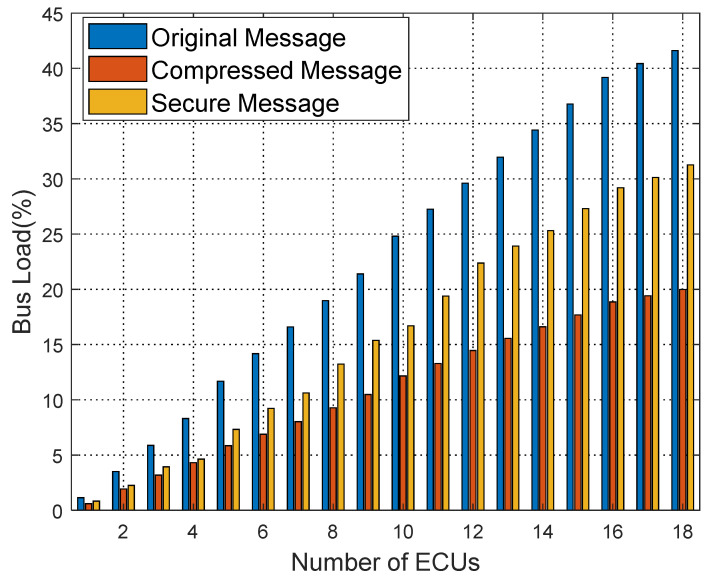
Comparison of Kia bus load ratio.

**Figure 17:**
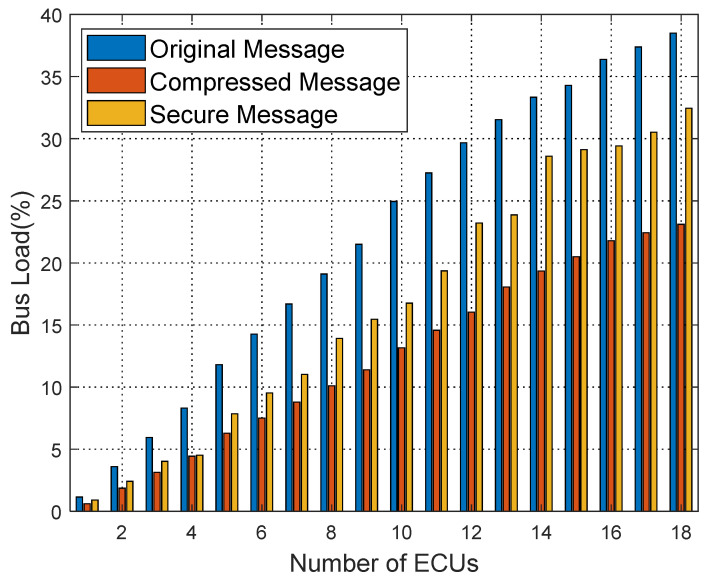
Comparison of bus load ratio of Honda.

**Figure 18:**
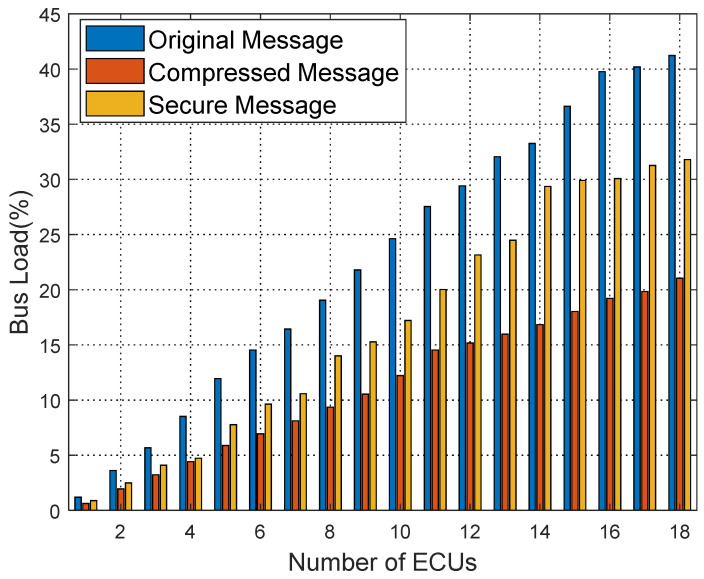
Chevrolet bus load rate diagram.

Figure 16, Figure 17 and Figure 18 show the bus load rate comparison for Kia, Honda, and Chevrolet vehicles’ raw, compressed, and secure messages. Specifically, for the Kia vehicle, the bus load rate is 41.61% during raw message transmission; using the compression algorithm, it reduces to 19.99%; with the proposed security protocol, the load rate is 31.26%. Similarly, for Honda and Chevrolet vehicles, the load rate decreases significantly with the proposed security protocol, averaging an 8.28% reduction in load rate from raw messages to secure messages.

## 5. Conclusions

The experimental model proposed here is more aligned with the actual environment of intelligent and connected vehicles. In the proposed security protocol, comprehensive evaluations are conducted on CAN bus signals with varying compression levels. This protocol uses the DBK dynamic key management algorithm for dynamic key generation, updating, and management, improving the bus’s security. Furthermore, AES-128 encryption and HMAC authentication algorithms are applied to execute corresponding security measures for messages with different compression levels, avoiding imbalances in bandwidth burdens. Each ECU node uses an independent key, updated regularly to increase security and prevent key leakage. Furthermore, the dynamic data compression approach increases the complexity of system analysis for adversaries, enhancing the system’s defensive robustness. These measures effectively prevent eavesdropping, brute-force, replay, and flood attacks, ensuring the security of the in-vehicle network. In future work, we will focus on optimizing key management strategies by exploring advanced techniques like IBE and ABE, while enhancing data compression efficiency and security. Particularly, we will investigate post-quantum cryptographic algorithms to counter potential quantum computing attacks, ensuring long-term security. Additionally, we aim to develop more robust authentication methods to strengthen data integrity and confidentiality.

## Figures and Tables

**Table 1:** Correlation analysis of CAN bus attack research.

Reference	Eavesdropping Attack	Brute-Force Attack	Replay Attack	Flood Attack
[10]	√			
[11]				√
[12]			√	
[13]	√			
[14]		√		
[15]	√			

**Table 2:** Correlation analysis of CAN bus security protocol types’ research.

Reference	Authentication Method	Bus Load Impact	Defense Attack Type
[16]	Identity Authentication Codes	High	Flood Attack
[18]	Entity Authentication	High	Replay Attack
[19]	Message Authentication	Low	Eavesdropping Attack
[20]	Hash Functions and Symmetric Encryption	High	Eavesdropping Attack
[21]	Lightweight Block Cipher	Medium	Replay Attack
[22]	Cryptographic Techniques	Low	Replay Attack
[23]	Centralized Authentication Method	Not specified	Brute-Force Attack
[24]	Key-Type Selection	Not specified	Eavesdropping Attack

**Table 3:** Correlation analysis of CAN bus data compression algorithms.

Reference	Signal Rearrangement	Reduce the Number of Bytes
[25]	√	
[26]	√	√

**Table 4:** Message matrix.

Bit 7	Bit 6	Bit 5	Bit 4	Bit 3	Bit 2	Bit 1	Bit 0
0	1	0	1	1	0	0	1

**Table 5:** Hardware usage table.

	Code	Inc. Data	RO Data	RW Data	ZI Data
Grand totals [bytes]	33,868	1308	1976	176	35,400
ROM totals [bytes]	33,868	1308	1976	176	0
Total RO size (flash) (code + RO data)					35.84 KB
Total RW size (RAM) (RW data + ZI data)					35.58 KB
Total ROM size (code + RO data + RW data)					36.02 KB

**Table 6:** Comparison of security protocols for CAN bus.

	Ref. [23]	Ref. [27]	Proposed
Authentication-key type	Global key	Pairwise key	Pairwise key
Long-term key	Yes	No	No
Authentication mode	Centralization	Distribution	Distribution
Message encryption	No	Yes	Yes
Network scalability	No	Yes	Yes
Key generation time	Not applicable	1 ms	0.0858 ms
KECU required	Not applicable	Yes	No

**Table 7:** Compression effect comparison.

ID	4bit-DA	DRC	Proposed
153	99.99%	99.99%	99.99%
18F	93.47%	94.16%	94.30%
329	90.72%	91.56%	92.18%
43F	81.42%	83.26%	87.29%
183	79.27%	81.25%	80.24%
580	92.17%	94.26%	94.45%
545	79.24%	80.53%	77.83%
316	73.87%	79.52%	80.67%
440	63.17%	65.23%	70.69%
1A4	79.66%	81.26%	87.50%
13A	78.56%	81.42%	95.11%
17C	76.21%	78.53%	88.76%
136	78.02%	81.77%	70.23%
378	75.50%	81.87%	77.18%
1ED	96.36%	96.36%	96.50%
1EF	95.51%	95.36%	96.26%
18E	99.99%	99.99%	99.99%
0F9	96.26%	95.97%	97.01%

**Table 8:** Compression time comparison.

Frequency (MHz)	4bit-DA (ms)	DRC (ms)	Proposed (ms)
30	0.0601	0.0613	0.0553
60	0.0312	0.0301	0.0268
90	0.0204	0.0212	0.0172
120	0.0168	0.0174	0.0138
168	0.0103	0.0217	0.0081
300	0.0073	0.007	0.0052
400	0.0047	0.0044	0.0039

## Data Availability

The original contributions presented in this study are included in the article. Further inquiries can be directed to the corresponding author.
